# Mechanistic yellow fever modelling under climate change in Brazil and beyond: Information gaps and future steps

**DOI:** 10.12688/wellcomeopenres.24901.1

**Published:** 2025-10-24

**Authors:** Livia Abdalla, Angélica S. da Mata, Keith J Fraser, Sally Jahn, Eduardo Krempser, Adriano Pinter, Alessandro Pecego Martins Romano, Antônio Ralph Medeiros-Sousa, Daniel Garkauskas Ramos, Helio Junji Shimozako, Luis Filipe Mucci, Luiz Antonio Costa Gomes, Luiz Carlos Junior Alcantra, Ramon Silva Oliviera, Rodrigo Otávio Pereira Sayago Soares, Vinicius Pereira Feijó, Douglas Augusto, Marcia Chame, Katy A M Gaythorpe

**Affiliations:** 1 Biodiversity, Wildlife Health Institutional Platform (PIBSS/Fiocruz), Fundacao Oswaldo Cruz, Rio de Janeiro, State of Rio de Janeiro, Brazil; 2 Physics, Universidade Federal de Lavras Departamento de Fisica, Lavras, State of Minas Gerais, Brazil; 3 Imperial College London School of Public Health, London, England, UK; 4 Veterinary School, Universidade de Sao Paulo, São Paulo, State of São Paulo, Brazil; 5 Secretariat of Health and Environmental Surveillance Ministry of Health, Secretariat of Health and Environmental Surveillance, Ministry of Health, Brasília, Brazil, Brasília, Brazil; 6 Postgraduate Program in Parasite Biology, Department of Microbiology and Parasitology, Federal University of Rio Grande do Norte, Federal University of Rio Grande do Norte, Natal, State of Rio Grande do Norte, Brazil; 7 Butantan Institute, São Paulo State Health Secretariat, sao paulo, São Paulo, Brazil; 8 Taubaté Regional Technical Group, Pasteur Institute, São Paulo State Health Secretariat, sao paulo, Taubaté, São Paulo, Brazil

**Keywords:** Yellow fever; Brazil; Mathematical modelling; Climate change; Surveillance

## Abstract

Yellow fever (YF) remains a significant public health threat in tropical regions, particularly in South America and Africa. The combined forces of climate change, land-use, urbanisation, globalisation, and insufficient surveillance and health infrastructure are driving the re-emergence and expansion of YF into new areas. While mathematical models have been used to estimate transmission risk, disease burden, and the impact of vaccination, there remains a crucial gap in mechanistic models that explicitly capture how climate and environmental changes directly influence YF transmission. To address this gap, we convened a workshop in Brazil as part of the Vaccine Impact Modelling Consortium’s Climate Change programme, bringing together national and international experts. The workshop aimed to present current modelling approaches, identify key knowledge gaps, and develop strategies to improve data collection and model applicability. Discussions highlighted major uncertainties regarding vectors, non-human primates, surveillance sensitivity, vaccination, and climatic and environmental drivers. This paper synthesises the outcomes of the workshop, including priority areas for future research and recommendations for advancing mechanistic YF modelling in the context of climate change, with a focus on both Brazil and broader tropical regions.

## Introduction

Yellow fever (YF) is a viral haemorrhagic fever endemic in tropical regions of South America and Africa. It was introduced to South America through the dessicated eggs of *Aedes aegypti* mosquitoes carried onboard ships transporting enslaved people and the first described outbreak in Brazil occurred in 1685 Chippaux and Chippaux [2018]. In addition to humans, the YF virus infects non-human primates (NHPs) including howler monkeys and marmosets, which act as a sylvatic amplifier host and source of infection to mosquitoes Medeiros-Sousa *et al.* [2024]. This makes eradication of the virus infeasible, with its occurrence in humans being primarily controlled through vaccination as well as through control of mosquito vectors Kurauchi *et al.* [2018]. Globalisation, ongoing population growth, and rapid urbanisation, combined with climate change and insufficient surveillance and public health infrastructure, including suboptimal vaccination coverages, are key drivers of the spread of YF virus into tropical and subtropical regions where mosquito vectors are present. In addition to effective surveillance, there is an urgent need to implement routine immunisation and targeted vaccination campaigns, alongside the development and application of diverse and innovative mosquito control strategies in endemic areas, to reduce the risk of future YF urban outbreaks and minimise its global impact Gianchecchi *et al.* [2022].

Mathematical modelling of YF has been used to assess areas of heightened risk of transmission de Thoisy *et al.* [2020], Kaul *et al.* [2018], Lim *et al.* [2025], Shearer *et al.* [2018], the overall burden Gaythorpe *et al.* [2021], Fraser *et al.* [2024], the dynamics and impact of vaccination both historically and in the future Fraser *et al.* [2024], Bonin *et al.* [2020], Handari *et al.* [2022], and the implications of climate and environmental change Gaythorpe *et al.* [2020], Hamlet *et al.* [2021], Kaye *et al.* [2024]. These studies vary in scope, question of interest, and data requirements; however, there are often assumptions that are required either to bridge missing information or as an implied component of the model structure.

In terms of modelling the implications of climate and environmental change on YF transmission dynamics and disease, approaches are more nascent. Whilst the link between YF occurrence and environmental conditions such as temperature, precipitation and vegetation is well discussed, the mechanisms of influence are less established, particularly for the sylvatic mosquitoes which drive the majority of transmission and the NHPs who are the sylvatic hosts. Current estimates of YF under climate change have focused on average future projections Gaythorpe *et al.* [2020], seasonality Hamlet *et al.* [2021] or variations in the population density and range of the urban vector, *Ae. aegypti* Kaye *et al.* [2024], Kraemer *et al.* [2015]. More nuanced interpretation and use of climate projections, coupled with deeper understanding of the mechanistic links between climate, environment and YF transmission are needed to provide detailed and actionable projections of YF in the future.

In an effort to deepen our insight and support more accurate projections, we held a workshop, ’International workshop on yellow fever modelling in Brazil to address challenges and scientific advancements in the context of climate change’, focused on YF modelling in Brazil as part of the Vaccine Impact Modelling Consortium’s (VIMC’s) programme on Climate Change vaccineimpact.org. The objectives of the workshop were to i) present current mathematical models of YF transmission, ii) identify gaps in knowledge to prioritize issues relating to the applicability of models, and iii) identify and develop strategies for obtaining data that can be used for the necessary advances in public policies for the prevention and control of YF. The workshop brought together researchers specialising in disease transmission dynamics from Brazilian research institutions and reference organizations, such as Fundação Oswaldo Cruz and the Ministry of Health, as well as international experts from Imperial College London, creating an environment conducive to knowledge exchange and the enhancement of modelling methodologies. The structure of the workshop and the key outcomes of these discussions are presented in the sections that follow.

## Main text

### Identification and prioritization of information gaps

**Figure 1.**
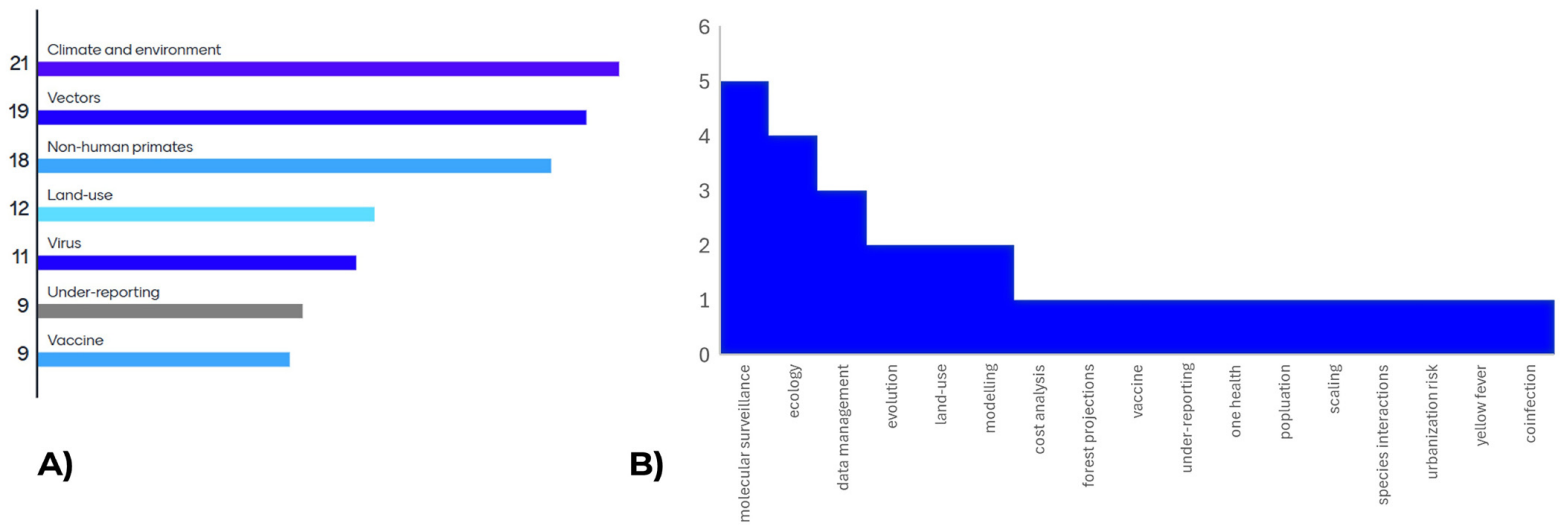
Results of survey of workshop attendees for **A**) prioritization of identified data gaps and **B**) additional suggestions for areas of uncertainty/ data gaps.

Through the first day of the workshop, attendees heard from a variety of modellers and yellow fever disease specialists who presented on the current situation of both mathematical modelling and epidemiology of yellow fever transmission in Brazil. These presentations from the Ministry of Health, Fiocruz and representatives from Imperial College London highlighted some of the current uncertainties and knowledge gaps in the yellow fever modelling space. To conclude the day, attendees were asked to prioritize the data gaps presented and identify any missed topics; the results are presented in Figure 1 and informed the workshop discussions for subsequent days.

The first day of presentations was facilitated by live simultaneous translation provided by two interpreters. Subsequent days of the workshop, which focused more on group discussion of the priority topics shown in Figure 1, were supported by live AI translation through Google meet where subtitles for the conversation were displayed on a screen. Whilst the AI translation is still relatively nascent, the process meant that there could be a smooth transition between discussions in Brazilian Portuguese and English. Also, as the AI translation required a microphone to be passed from one speaker to the next, this meant that each individual was given a platform to comment without interruptions. Further, immediate transcription meant that each person’s contributions could be collated each day. Finally, as each priority topic was examined, speakers were invited to comment from all institutions to ensure that everyone had had a chance to contribute.

### Climate and environment

Climate, weather, current land use and land cover are influential on the hosts and vectors of YF either through their dynamics, through their habitat, or through their behaviour. Mosquitoes are ectothermic and breed in water, and thus their lifespan, biting habits and reproduction are all dictated by the climate and environment in which they live Reinhold *et al.* [2018]. Even their range and dispersal may be affected by wind patterns Atieli *et al.* [2023]. NHPs require certain habitat or vegetation types as well as access to sustenance and water to live; yet, conditions that threaten resources such as water and food will lead to expanding ranges of mobility and to increased species dominance (with reduced richness), potentially leading to increased confrontations with humans Kaisin *et al.* [2021], Estrada *et al.* [2020]. Finally, extreme weather events can increase the risk and spread of arbovirus transmission to new areas and affect the delivery of healthcare services to humans and thus the overall success of intervention strategies such as vaccination Dewi *et al.* [2024]. Recent studies have investigated how vegetation, land cover, climate, and host population contribute to the timing and distribution of reported yellow fever (YF) cases and other vector-borne diseases Gianchecchi *et al.* [2022]. Vegetation type, habitat fragmentation, and land cover appear to play a role in shaping YF transmission patterns. Although human activity is known to alter vegetation over time, potentially influencing disease emergence, this is relatively understudied. Nevertheless, habitat fragmentation may affect sylvatic hosts by increasing their contact with humans through behavioural changes, or by weakening their immune systems due to stress, making them more susceptible to infection White and Razgour [2020]. Furthermore, fragmentation can result in a reduction in the richness of host and vector species, with a consequent increase in the dominance of species involved in pathogen transmission. Vegetation heterogeneity may also influence mosquito vector dynamics by increasing opportunities for human exposure to the sylvatic cycle or by creating conditions that favour adaption to anthropophilism by mosquito species in fragmented habitats Hamlet *et al.* [2021], Wilk da Silva *et al.* [2023], Faust *et al.* [2018]. Weather station and gauge data, reanalysis and observational datasets, and outputs from Global Climate Models (GCMs) are essential tools for understanding weather and the climate system, as well as for projecting its evolution under various emissions scenarios. The use of weather and climate data, along with climate model outputs, is based on a well-established scientific discipline that provides essential inputs for health-related impact models. Model outputs from inter-comparison projects like CMIP produced within the World Climate Research Programme (WCRP) Masson-Delmotte *et al.* [2021], Eyring *et al.* [2016], along with projections provided that are derived from them using statistical and dynamical bias-correction and downscaling methods (e.g., Gergel *et al.* [2024]; Thrasher *et al.* [2022]; Warszawski *et al.* [2013]) are frequently used in climate impact research to assess relationships under both historical and present-day climate patterns (e.g., such as in attribution studies that aim to quantify the impact of historical human-caused climate change on disease burden and outbreaks), as well as to assess how these relationships may evolve under future climate change conditions (e.g., projecting trends in disease occurrence under future climate change scenarios) Colón-González *et al.* [ 2021, 2023]. However, these data products are often unwieldy or require specialist expertise to utilize. Choosing the appropriate data product is complex, as each comes with its own limitations and specific scope of application. This requires a thorough understanding of the data generation, modelling and processing approaches, as well as the experience in appropriate use, such as knowing which bias-correction and downscaling techniques have been applied or are still needed, or validating projections with an appropriate data source for the geography in question. Furthermore, if any of these provided pre-processed climate projection datasets do not fully meet the specific needs of a study — such as when researchers want to include additional variables or address further uncertainties related to model spread or scenarios for a particular region — it becomes necessary to perform bias-correction and downscaling independently. This increases the complexity and creates a higher barrier to integrating climate model outputs. Furthermore, the choice of observational datasets used as reference climatologies not only influences the bias-correction and downscaling of climate model outputs but can also directly affect the assessment of relationships between weather, climate, and critical factors influencing health outcomes, and thereby the driving of impact models Baker *et al.* [2016], Jahn *et al.* [2025a]. It is therefore essential to account for the fact that observational data carry their own uncertainties and are often best regarded as informed approximations rather than absolute truth Jahn *et al.* [2025b], Lavers *et al.* [2022], Hassler and Lauer [2021].

To resolve the issue of appropriate data use and extraction, standardized weather and climate information is required in an accessible format alongside a set of modelling assumptions and caveats written in plain language. In practice, data products have sometimes been selected based on availability, format convenience, or familiarity with the provider — frequently relying on datasets that are based on simple bias-correction and downscaling methods (e.g., WorldClim Fick & Hijmans [2024]). While these approaches can capture basic climate change signals, they are often insufficient for detecting critical future shifts in seasonality or extremes, which are often key drivers of disease outbreaks and health burdens. While climate and environmental data have already been used to effect in health impact studies (e.g., de Souza & Weaver [2024]; Gaythorpe *et al.* [2020]; Ryan *et al.* [2019]), less is mentioned on the data extraction, validation and appropriate use. If these issues were addressed, it would be feasible to update the models estimation and simulation in a more timely manner whilst exploring the possibility of different future scenarios. Finally, clear and transparent communication of the uncertainties is needed to delicately balance the issue of overly dramatic or subdued projections of future vector behaviour and vector occurrence. This highlights the urgent need for clearer guidelines, accessible tools, and coordinated initiatives to support the effective integration of environmental and climatic data into health research and decision-making.

### Vector

YF is a mosquito-borne disease in both the sylvatic and urban cycles Monath [2001]. The mosquitoes involved in sylvatic transmission include the *Haemagogus* and *Sabethes* genera Cruz *et al.* [2023], de Abreu *et al.* [2019], de Oliveira *et al.* [2023]. In the African endemic region, *Ae. aegypti* is the main vector of the urban cycle. The last urban yellow fever transmission by *Ae. aegypti* in Brazil was recorded in 1942. Since then, infection in Brazil is presumed to only occur through sylvatic spillover and thus the changing feeding habits of *Haemagogus* and *Sabethes* are critical to the occurrence of infection in humans Monath [2001]. The key data gaps and obstacles revolve around vector distribution, including habitat range and movement range, their response to the human and natural environment, and vector competence or persistence of infection. Often vectors are collected in specific setting and shared with reference laboratories but the ensuing data on the proportion of mosquito pools that test positive is not systematized downstream for use in other activities Foley *et al.* [2009]. Authorship and ownership of data, along with recognition of effort and attribution in data production, is a key consideration and, when not handled transparently, can be a barrier to data access and sharing Cuff *et al.* [2024]. There are notable species and geographic biases in the existing literature, particularly regarding research into thermal response of vectors which often focuses on *Ae. aegypti* Mordecai *et al.* [2019]. Finally, in order to appropriately identify collected species of vectors, a sufficient level of expertise in entomology is required which calls for retaining and supporting the capacity within laboratories and elsewhere in the data pipeline. In this topic, data is currently available. However, there are significant barriers to use of the data and a lack of systematization and network to share information whilst retaining appropriate ownership and/or usage licenses. To address, leveraging existing data collection and sharing facilities to collate and standardize vector occurrence, positivity and distribution is vital. Beyond Brazil, optimising the use of samples and vectors that are routinely collected, such as through broad diagnostics and species identification, can provide critical information for informing models of species distribution and positivity. Information on vector occurrence has been used in modelling to provide an estimate of species distribution Kraemer *et al.* [2015]. Thus, updates to the occurrence and location of vectors by species have a clear use case in species distribution and projection. Similarly, studies have utilized laboratory examinations of the thermal response of vectors such as *Ae. aegypti* Gaythorpe *et al.* [2020], Mordecai *et al.* [2019]. However, as *Ae. aegypti* has not been found to contribute to current transmission cycles in Brazil, and as the related *Ae. albopictus* does not play a significant role, more information is needed on how sylvatic mosquitoes are affected by weather and climatic variation Damasceno-Caldeira *et al.* [2023], Celone *et al.* [2022], Alencar *et al.* [2022]. Additionally critical for the sylvatic cycle is the biting preferences of these mosquitoes and whether this is influenced by human or NHP behaviour Mucci *et al.* [2015]. The cross-immunity and cross-infection with other arbovirus has been found to amplify the competency of vectors and such an effect would be highly influential in any epidemiological modelling Öhlund *et al.* [2019]. Lastly, the movement of vectors and radius of effect is critical for examining areas of higher risk, Abreu *et al.* established that the majority of cases in Brazil occurred up to 11 km from infected NHPs; such an analysis in other geographies could refine existing areas of YF risk de Abreu *et al.* [2022].

### Non-human primates

Non-human primates (NHPs) are the main hosts for transmission in South America. Originally introduced from Africa, YF in South America severely negatively impacts NHP populations due to a high mortality rate in some species de Azevedo Fernandes *et al.* [2021], Nederlof *et al.* [2025]. Whilst it is acknowledged that the NHP population is vital for amplifying YF virus availability in the environment, there are still notable unknowns in modelling NHPs. Monitoring epizootic cases among NHPs thus serves not only to elucidate these ecological dynamics, but also as a critical early warning system for human YF virus transmission, prompting coordinated actions by surveillance to mitigate viral spread Romano *et al.* [2014], Goes de Jesus *et al.* [2020].

There are 131 NHP species in Brazil, and whilst not all of these are affected by YF, there are key differences in the demographics and epidemiology of primate species that can carry the YF virus and serve as a source of infection Nederlof *et al.* [2025], Farias *et al.* [2024]. Two priority genera are *Alouatta* and *Callithrix*, both of which are susceptible to YF infection and suffer a high mortality rate from it, but also have substantially different lifespans and may be reported at different rates due to their sizes and behaviours. Understanding the demography of NHPs as well as their different experiences and outcomes of YF infection is key to understanding how transmission cycles can continue in the environment.

Some estimates exist of both differential yellow fever susceptibility, viral load, and mortality rates in NHPs in Brazil de Azevedo Fernandes *et al.* [2021], and their distribution Culot *et al.* [2019], but there are still uncertainties in population distribution, density and movement. Linking existing information is key, for example by examining Ministry of Health reports on deceased primates and whether they were positive for YF, the huge citizen science project SISS-Geo Chame *et al.* [2019], and estimates of NHP species distribution from Salve, https://salve.icmbio.gov.br/#/, or other datasets Costa-Araújo *et al.* [2024]. This would allow projections of population sizes as well as the cycle of transmission in differing geographic regions. Efficient testing of NHPs in the environment, potentially through a rapid diagnostic test, would allow a quick assessment of the local infection pressure of the sylvatic hosts. Furthermore, whilst there are reports of NHP adaptive behaviours due to weather related food and water scarcity, quantifying how an extreme weather event such as drought will affect NHP mobility is complex.

If data were available to robustly estimate a model of NHP demographics and epidemiology, we could explore a number of critical questions. Firstly, local transmission potential could be assessed, as well as factors contributing to heightened risk of outbreaks. This could pre-empt the usual practise of using NHP mortality as a potential sentinel of YF transmission, thus improving early warning for humans and leading to NHP conservation. In addition, other control strategies can be assessed, such as NHP vaccination in captivity. Such an assessment would currently be hampered by lack of knowledge of NHP population levels, and lack of understanding of how different lifespans and infections between species can affect the sustainability of transmission in a certain geography. However, studies exist on safety of the YF vaccine in NHPs Tavares da Silva Fernandes *et al.* [2021], de Paula *et al.* [2025], de Miranda *et al.* [2022]. Similarly, there are key concerns with the vaccination of NHPs particularly around virus evolution and their use as sentinels Saad and Chiaravalloti-Neto [2024]. Understanding how the NHP may react to vaccination, could help project the risks of virus adaptation; and being able to estimate existing infection levels, could provide insight on the likelihood of primate mortality and thus use as a sentinel of YF Tavares da Silva Fernandes *et al.* [2021], de Paula *et al.* [2025]. Finally, data collection on NHP movement and behaviour in both times of calm and times of extreme weather, or other disruption, may help project how waves of YF may propagate spatially. However, the possibility that other species are somehow involved in sylvatic cycles, such as small arboreal mammals, should not be ruled out.

### Surveillance sensitivity

Effective modelling is grounded in robust data with a clear link between the process of data collection and the interpretation within the model. Surveillance, whilst unlikely to capture all cases or occurrences, particularly in wildlife infections, is key to obtaining data, and the quality of its completeness Andrade *et al.* [2022]. Yet, surveillance sensitivity varies over time, geography and species examined. If this is not taken into account in modelling, it can bias the estimates of epidemiological parameters and give a false impression of burden Gamado *et al.* [2014]. Surveillance effort is notoriously difficult to assess as a lack of report could mean either absence or a missed notification. Additionally, geographical surveillance differences can lead to diplomatic tensions if one region is seen to be less effective at surveillance. Often there are no estimates on how reporting has changed over time, or how one surveillance or health system can be compared to another. It is important to emphasise that in the case of yellow fever, ongoing surveillance is influenced by the occurrence of outbreaks of other arboviruses with a major human impact, such as Zika, dengue, and chikungunya, since the same surveillance and assistance services are responsible for all of them.

Some modelling studies have attempted to quantify surveillance variation, or examine the sensitivity of modelled estimates of epidemiological parameters to differing assumptions on surveillance effort. The Global arbovirus initiative has collated information across arboviruses and other vaccine preventable diseases to aid in projecting surveillance variations Lim *et al.* [2025]. Similarly, through the estimation process of many YF models, there is also some assumption on the process of observation when using case and death notification data, whether implicitly or explicitly stated Fraser *et al.* [2024], Gaythorpe *et al.* [2021]. As such, comparing information from different sources can help to estimate surveillance intensity and sensitivity through the model estimation framework. Incorporating a grounded estimate of surveillance sensitivity through time or space could allow a more specific estimate of key epidemiological parameters and thus disease burden. Furthermore, an effective threshold of notification can be described to aid in early warning systems and initial planning of outbreak responses.

### Vaccine

Vaccination is the primary form of control for YF outbreak response and routine management of YF risk. Conversely, understanding who may have been missed by vaccination can inform who may be at an increased risk of infection and thus a priority target for interventions. Whilst the WHO guidance is that one dose of YF vaccination provides lifelong protection, there are some studies examining potential waning of immunity which would affect the population levels of protection Schnyder *et al.* [2024]. Establishing pre-existing coverage relies on consistent estimates of routine coverage achieved and doses given in campaign activities for prior years as well as accurate estimates of population sizes Gaythorpe *et al.* [2024]. Further, as the estimates of both doses and population can be affected by short and long term migration, estimates of human mobility are also ideally needed. There are existing gaps in doses provided, particularly for historical activities, and population estimates are often projected from historical census data which varies in completeness and recency. Finally, there are population sub-groups who are often missed in vaccination activities such as working age men, who are also disproportionately represented in YF case data Feijó *et al.* [2024], McClure *et al.* [2025]. Retaining and monitoring consistent records of YF vaccination activities, as well as whether individuals move to other regions, may provide more robust estimates of coverage levels in a particularly geographic area. Linking datasets of activities with robust estimates of populations either collected directly through census’ or through separate proxies such as voter rolls, would allow sense checking of projected coverage estimates. Finally, followup studies in high coverage settings are needed to confirm estimates of waning immunity in high transmission settings to build on existing studies Schnyder *et al.* [2024].

Including accurate vaccination information in the models of burden is critical to provide a realistic picture of both immunity and influence any projected impact of future vaccination activities. Understanding the profile of immunity over time and in different settings can allow more nuanced examination of the impact of fractional or booster vaccination. Further, identifying subpopulations who are at particular risk of infection can help optimize delivery of interventions.

To illustrate the importance of understanding vaccination gaps in Brazil, recent studies have shown that antibodies generated by the yellow fever vaccine may be less effective against newly circulating Brazilian strains. This reduced potency is linked to specific genetic differences in the virus, underscoring the urgent need to reassess vaccine coverage, immunity levels, and surveillance strategies in the region Haslwanter *et al.* [2022]. To exemplify additional challenges related to YF vaccination, one important risk involves unvaccinated travellers who may introduce the virus into non-endemic countries. Moreover, because the YF vaccine is a live-attenuated virus, it is contraindicated for immunocompromised individuals, such as those living with HIV or taking immunosuppressive medications. In these cases, vaccination decisions must consider several factors, including the traveller’s age, destination, medical history, and immune status Lown *et al.* [2012]. Finally, beyond these challenges, limited global vaccine supply remains a concern. Although fractional dosing has been used as a temporary strategy to extend coverage during emergencies, it may not provide long-term protection, highlighting the urgent need to strengthen routine vaccination programs and increase vaccine availability Martins *et al.* [2013], Roukens and Visser [2019], Vianna *et al.* [2024].

### Virus

Understanding virus dynamics, evolution potential and characteristics is key to assessing current and future risks. Whilst the use of genomic epidemiology has helped to establish the origins Mir *et al.* [2017], spatial corridors of YF in Brazil Giovanetti *et al.* [2023], and re-emergence Andrade *et al.* [2021], there are still unknowns and limitations concerning the virus. Conversely, there are opportunities with the relatively recent advances in genomics to embed additional information into mathematical models, enriching the potential of both Stockdale *et al.* [2022]. Apart from the benefits of increased sequencing and testing, i.e. the call for more information in general, there are specific connections that are not well understood between virus diversity and host characteristics. For example, how YF may compare to other similar viruses such as dengue in terms of stability- it appears that YF is more stable, is this a product of less transmission or otherwise? Similarly, would changes in environmental factors such as temperature and precipitation have implications for the mutation rate of the virus Hill *et al.* [2022]. Furthermore, it is less clear how virus evolution changes with the myriad of hosts and vectors involved in YF virus transmission. Increasing genomic sequencing and sharing of data and results in appropriate databases is opening up possibilities for understanding viral dynamics in Brazil and elsewhere Andrade *et al.* [2022]. Addressing issues surrounding data authorship, acknowledgement and barriers to sharing can help with dissemination and facilitate the generation of open and reproducible model outcomes. Further building expertise in pipeline from sequencing to analysis is essential to utilise the possible developments in this field and its integration with diseases surveillance and modelling more generally.

Modelling can benefit from genomic information through reinforcing existing activities such as parameter estimation- genomic data generally providing a richer picture of the dynamics and transmission to date than might otherwise be captured in case counts. However, it can go further by examining more detailed stratifications of host or virus characteristics and where they may have originated from. When linked with appropriate metadata, genomic sequencing can provide a rich resource and potentially benefit both retrospective analyses and future forecasting Stockdale *et al.* [2022].

## Conclusions

Through the workshop and following work, key areas of data uncertainty and outstanding questions around mathematical modelling of yellow fever transmission, particularly in the context of climate change, were identified. We have examined the current context and suggested ways forward to address data gaps and barriers to access. The Recommendations box details both short-term and longterm considerations regarding yellow fever data, and this may be applicable more broadly both geographically and to other epidemiological data. The workshop allowed us to discuss issues, and in many cases find that there were potential solutions within reach either through existing datasets or by combining a variety of information within the modelling frameworks to gain insight on yellow fever epidemiology. As we increasingly see re-emergence and spread of YF outbreak occurrence facilitated by human and climatic change, we see a huge amount of potential for linking people and data to gain insight and improve both public and animal health.

## Disclaimer

The views expressed in this article are those of the author(s). Publication in Wellcome Open research does not imply endorsement by the Wellcome Trust.

## Supplementary information

### Extended data

*Repository name: [mechanistic_yf_workshop_SI]* https://github.com/mrc-ide/mechanistic_yf_workshop_SI/. ( Gaythorpe & Fraser)

*The project contains the following extended data:*

• *Mechanistic yellow fever modelling under climate change in Brazil and beyond - Supplementary Information.pdf which provides supplementary figures of wordclouds of priority areas of uncertainty per topic, links to resources and summary tables of data gaps identified through the workshop*.

*Data are provided under a CC0-1.0 license.*

## Recommendations

### Short-term

- Establish current status of available datasets and methods/routes to extraction of positive and negative data
- Ensure curation/recognition of stakeholders and maintain dialogues with data producers and data consumers
- Provide motivating example of subnational modelling where data is currently available

### Long-term

- Expand and leverage existing initiatives to:
  – Generate and collect missing data
  – Integrate existing data with other linked information
  – Utilize analysis frameworks
- Create a data environment and analysis framework that leverages existing initiatives.
- Integrate appropriate data standards to ensure consistency between disparate activities
  – Encourage the use of metadata and integration into central databases.
  – Develop a data dictionary and bibliography to support standardization across platforms.
  – Ensure clear documentation and structured storage of data for accessibility and long-term use (curation, updating, and maintenance of the data)
- Ensure a transparent and equitable approach to authorship on outputs from data collection inputs
- Expand regional modelling to national projections
- Progress beyond current geographical and epidemiological scope to support efforts in other disease areas and countries
