# Supplementary material for: Mechanistic yellow fever modelling under climate change in Brazil and beyond: Information gaps and future steps
Source: Wellcome Open Res. 2026 Mar 13;10:596. Originally published 2025 Oct 24. [Version 2] doi: 10.12688/wellcomeopenres.24901.2 (PMC13040231; doi:10.12688/wellcomeopenres.24901.2)
Supplement: Peer review history

# Reviewer report: Reviewer response for version 2

**DOI:** 10.21956/wellcomeopenres.28695.r150918

**Published:** 2026-03-31

**Authors:** Preety Kalra

**Affiliations:** 1 Mathematics and Statistics, Lovely Professional University School of Chemical Engineering and Physical Sciences, Phagwara, Punjab, India

The proposed work should incorporate the following points to sets a strong research agenda with clear recommendations.

- Add 1-2 citations on debated topics e.g., flavivirus interference contradicting vector amplification claims.
- Explicitly discuss workshop biases e.g., Brazil-heavy participants, nascent AI translation and generalizability to Africa.
-  Include raw survey data or scales from Figure 1 in supplements for transparency.

Does the article adequately reference differing views and opinions?

Yes

Are all factual statements correct, and are statements and arguments made adequately supported by citations?

Yes

Is the Open Letter written in accessible language?

Yes

Where applicable, are recommendations and next steps explained clearly for others to follow?

Yes

Is the rationale for the Open Letter provided in sufficient detail?

Yes

Reviewer Expertise:

modelling in epidemiology

I confirm that I have read this submission and believe that I have an appropriate level of expertise to confirm that it is of an acceptable scientific standard.

# Reviewer report: Reviewer response for version 2

**DOI:** 10.21956/wellcomeopenres.28695.r150724

**Published:** 2026-03-20

**Authors:** Sakarn Charoensakulchai

**Affiliations:** 1 Mahidol University, Salaya, Nakhon Pathom, Thailand

Thank you for the revision. The article has added a substantial details as suggested by reviewers and the reviewers’ comments are well-responded by the authors. I have no further comments.

Does the article adequately reference differing views and opinions?

Yes

Are all factual statements correct, and are statements and arguments made adequately supported by citations?

Yes

Is the Open Letter written in accessible language?

Yes

Where applicable, are recommendations and next steps explained clearly for others to follow?

Yes

Is the rationale for the Open Letter provided in sufficient detail?

Yes

Reviewer Expertise:

travel medicine, tropical medicine, one health

I confirm that I have read this submission and believe that I have an appropriate level of expertise to confirm that it is of an acceptable scientific standard.

# Reviewer report: Reviewer response for version 1

**DOI:** 10.21956/wellcomeopenres.27428.r137355

**Published:** 2025-11-22

**Authors:** Sakarn Charoensakulchai

**Affiliations:** 1 Mahidol University, Salaya, Nakhon Pathom, Thailand

Thank you for invitation for reviewing this open letter. This open letter is about the outcomes of the workshop on yellow fever (YF) modelling in Brazil to address the challenges and advancement regarding to climate change, which yield intersting outputs. Climate change is an urgent issue that can result in changing of the ecology of several vector-borne diseases. Together with gloablisation, the threat of YF is imminent. These are my comments:

 1. I would recommend changing the title to: Information gaps and future steps for yellow fever modelling under climate change in Brazil and beyonf: output from an international workshop, or something similar, to emphasize that the outputs are from the workshop and be more intriguing.

 2. It would be good for readers if an example of events in which climate and environmental change has affected the distribution and ecology of YF transmission, such as the emergence of YF in coastal areas of Brazil during 2016 outbreaks and beyond. 

 3. In 'Vector' section first sentence, YF can also have savannah cycle in Africa.

 4. It is still controversial about cross-immunity and cross-infection with other arbovirus will amplify the competence of the vector. Some studies in Asia postulated that dominance of other flaviviruses in some areas interfered with the vector mosquitoe cells' ability to receive another type of flavivirus. ( https://doi.org/10.1155/2013/905043)

 5. Most of the times, the reports of YF outbreaks in non-human primates are usually among the fatal cases, which could be the tip of the iceberg. Given that not all of YF cases are fatal, the overall number of infected non-human primates could be several times higher than the reported fatal cases. Immunity status against YF in non-human primates can also be importatnt. It is difficult to obtain this data from wild non-human primates, but this number could be crucial for modelling the transmission patterns in the wild.

 6. Is YF vaccine included in the national EPI program? Are there differences in the policy of YF vaccine in EPI between each federal states? And how much coverage of the vaccine is in each federal state? These data would be importatnt for assessing the at-risk population. 

 7. Addressing the requirements and robustness of checking for the proof of YF immunization in travellers could be important for preventing the spread of YF to other countries, and within country (in case a traveller acquired YF from one region and travel to another non-endemic region within Brazil).

Does the article adequately reference differing views and opinions?

Yes

Are all factual statements correct, and are statements and arguments made adequately supported by citations?

Yes

Is the Open Letter written in accessible language?

Yes

Where applicable, are recommendations and next steps explained clearly for others to follow?

Yes

Is the rationale for the Open Letter provided in sufficient detail?

Yes

Reviewer Expertise:

travel medicine, tropical medicine, one health

I confirm that I have read this submission and believe that I have an appropriate level of expertise to confirm that it is of an acceptable scientific standard.

# Response

**Published:** 2026-03-03

**Authors:** K Gaythorpe

**Affiliations:** Imperial College London School of Public Health, London, England, UK

*Many thanks for your positive review and comments. We have responded to these below and in 'version 2' of the manuscript.*

 Thank you for invitation for reviewing this open letter. This open letter is about the outcomes of the workshop on yellow fever (YF) modelling in Brazil to address the challenges and advancement regarding to climate change, which yield intersting outputs. Climate change is an urgent issue that can result in changing of the ecology of several vector-borne diseases. Together with gloablisation, the threat of YF is imminent. These are my comments:

 1. I would recommend changing the title to: Information gaps and future steps for yellow fever modelling under climate change in Brazil and beyonf: output from an international workshop, or something similar, to emphasize that the outputs are from the workshop and be more intriguing.

***Thank you for this suggestion. As the current title was agreed with all workshop participants, we have retained this for now.***

 2. It would be good for readers if an example of events in which climate and environmental change has affected the distribution and ecology of YF transmission, such as the emergence of YF in coastal areas of Brazil during 2016 outbreaks and beyond.

***Thank you, we have addressed this in a previous response of reviewer 1 regarding the risk of urban YF.***

 3. In 'Vector' section first sentence, YF can also have savannah cycle in Africa.

***We have updated this to “YF is a mosquito-borne disease in both the sylvatic and urban cycles Monath [2001]. The mosquitoes involved in sylvatic transmission include the Haemagogus and Sabethes genera Cruz et al. [2023], de Abreu et al. [2019], de Oliveira et al. [2023] in South America. In the African endemic region, Ae. aegypti is the main vector of the urban cycle, and there can be an additional, savannah cycle Monath [2001].”***

 4. It is still controversial about cross-immunity and cross-infection with other arbovirus will amplify the competence of the vector. Some studies in Asia postulated that dominance of other flaviviruses in some areas interfered with the vector mosquitoe cells' ability to receive another type of flavivirus. (https://doi.org/10.1155/2013/905043)

***Thank you, this comment is noted.***

 5. Most of the times, the reports of YF outbreaks in non-human primates are usually among the fatal cases, which could be the tip of the iceberg. Given that not all of YF cases are fatal, the overall number of infected non-human primates could be several times higher than the reported fatal cases. Immunity status against YF in non-human primates can also be important. It is difficult to obtain this data from wild non-human primates, but this number could be crucial for modelling the transmission patterns in the wild.

***Thank you for this point. We have discussed the variability between species and surveillance sensitivity in the manuscript. We acknowledge that under-reporting is an issue that needs to be addressed in modelling and highlight some examples of where this has been approached, and future directions in this area.***

 6. Is YF vaccine included in the national EPI program? Are there differences in the policy of YF vaccine in EPI between each federal states? And how much coverage of the vaccine is in each federal state? These data would be importatnt for assessing the at-risk population.

***We agree and discuss the need for comprehensive vaccination coverage information for understanding current and future immunity profiles.***

 7. Addressing the requirements and robustness of checking for the proof of YF immunization in travellers could be important for preventing the spread of YF to other countries, and within country (in case a traveller acquired YF from one region and travel to another non-endemic region within Brazil).

***Thank you for this point. Previous work has examined the risk of exportation of YF to other countries from Brazil, Dorigatti et al. 2019, and from other countries, Cracknell-Daniels et al. 2021.***

# Reviewer report: Reviewer response for version 1

**DOI:** 10.21956/wellcomeopenres.27428.r137356

**Published:** 2025-11-11

**Authors:** Cesar Cabezas Sanchez

**Affiliations:** 1 INSTITUTO NACIONAL DE SALUD, Lima, Peru

**Main comments**

1. The manuscript is presented as an *Open Letter* but functions as a hybrid between a mini-review and a workshop report. I suggest clarifying in the Introduction that it summarises an expert workshop and proposes a research agenda for yellow fever modelling under climate change, using Brazil as a case study. Please also specify which insights are Brazil-specific and which are transferable “beyond” Brazil.
2. The workshop methods underlying the prioritisation (Figure 1) need more detail. A short “Workshop methods” subsection should describe number and profile of participants, how priorities were collected (survey, voting, consensus) and what scale or ranking system was used. This would give more weight to the prioritised gaps.
3. The different domains (climate/land-use, vectors, non-human primates, surveillance, vaccination, virus/genomics) are well discussed but somewhat in parallel. A simple conceptual figure linking **climate/land-use → vectors/NHPs → viral dynamics → surveillance signals → vaccination decisions → modelling outputs**, with key data gaps marked at each step, would greatly help readers.
4. The link between data gaps and concrete public health decisions could be made more explicit. At the end of each subsection, one or two sentences could indicate how failing to improve those data constrains decisions (e.g. where to prioritise vaccination, where to strengthen NHP or entomological surveillance).
5. Given the emphasis on climate change and *Aedes* expansion, a short paragraph explicitly discussing current and future **risk of urban yellow fever** in Brazil would be valuable, integrating evidence and expert opinion on how climate and vector ecology might alter that risk.
6. In the virus/genomics section, it would help to be more explicit about how genomic data would feed mechanistic models (e.g. refining parameters such as number of introductions, dispersal speeds, transmission duration, R₀/Rₑ in different ecological contexts, and how phylogeography can be coupled to spatial risk models).

Does the article adequately reference differing views and opinions?

Partly

Are all factual statements correct, and are statements and arguments made adequately supported by citations?

Yes

Is the Open Letter written in accessible language?

Yes

Where applicable, are recommendations and next steps explained clearly for others to follow?

Partly

Is the rationale for the Open Letter provided in sufficient detail?

Partly

Reviewer Expertise:

Tropical diseases

I confirm that I have read this submission and believe that I have an appropriate level of expertise to confirm that it is of an acceptable scientific standard, however I have significant reservations, as outlined above.

# Response

**Published:** 2026-03-03

**Authors:** K Gaythorpe

**Affiliations:** Imperial College London School of Public Health, London, England, UK

*Many thanks to Dr Cabezas Sanchez for the helpful comments and thoughtful review. We have responded to the points raised below and in 'version 2' of the manuscript.*

 The manuscript is presented as an Open Letter but functions as a hybrid between a mini-review and a workshop report. I suggest clarifying in the Introduction that it summarises an expert workshop and proposes a research agenda for yellow fever modelling under climate change, using Brazil as a case study. Please also specify which insights are Brazil-specific and which are transferable “beyond” Brazil.

***Thank you, we have included a sentence at the end of the introduction to clarify the intent and added further to the conclusion of the transferability of the conclusions.***

 The workshop methods underlying the prioritisation (Figure 1) need more detail. A short “Workshop methods” subsection should describe number and profile of participants, how priorities were collected (survey, voting, consensus) and what scale or ranking system was used. This would give more weight to the prioritised gaps.

***We have included a further section including numbers of participants, disciplines and process for identifying gaps.***

 The different domains (climate/land-use, vectors, non-human primates, surveillance, vaccination, virus/genomics) are well discussed but somewhat in parallel. A simple conceptual figure linking climate/land-use → vectors/NHPs → viral dynamics → surveillance signals → vaccination decisions → modelling outputs, with key data gaps marked at each step, would greatly help readers.

***Thank you for this comment. We have included a new figure, figure 2, which links the subjects and brings out some of the major challenges with each.***

 The link between data gaps and concrete public health decisions could be made more explicit. At the end of each subsection, one or two sentences could indicate how failing to improve those data constrains decisions (e.g. where to prioritise vaccination, where to strengthen NHP or entomological surveillance).

***We have now included one-two sentences at the end of each section to emphasise what the negative outcomes are of not addressing the issues from a modelling perspective, and how this may translate into public health decisions*.**

 Given the emphasis on climate change and Aedes expansion, a short paragraph explicitly discussing current and future risk of urban yellow fever in Brazil would be valuable, integrating evidence and expert opinion on how climate and vector ecology might alter that risk.

***Thank you for this important suggestion. We agree that a clearer discussion of the current and future risk of urban yellow fever in Brazil- considering climate change and Aedes expansion- adds value to the manuscript. We have now incorporated a concise discussion in the Vector section  that synthesizes recent entomological and genomic evidence, as well as landscape and climate-sensitive modeling, to show that the risk of urban YF re-emergence in Brazil is tangible, though not yet widely detectable.***

 In the virus/genomics section, it would help to be more explicit about how genomic data would feed mechanistic models (e.g. refining parameters such as number of introductions, dispersal speeds, transmission duration, R₀/Rₑ in different ecological contexts, and how phylogeography can be coupled to spatial risk models).

***Thank you for this point, we have expanded the potential use in modelling. The integration of epidemiological and genetic data and knowledge is a vast and promising field that allows for the improvement of parameters and methodological approaches for the construction of mechanistic models. We will elaborate on this point in the section, including references such as https://doi.org/10.1093/molbev/msz106 and https://www.nature.com/articles/s41564-022-01233-6***
